# The association of metformin and aspirin intake with mammographic breast density: A cross-sectional study

**DOI:** 10.22088/cjim.14.4.741

**Published:** 2023

**Authors:** Bita Eslami, Ramesh Omranipour, Arvin Arian, Leila Bayani, Mahboubeh Abedi, Sadaf Alipour

**Affiliations:** 1 Breast Diseases Research Center, Cancer Institute, Tehran University of Medical Science, Tehran, Iran; 2 Department of Surgical Oncology, Cancer Institute, Tehran University of Medical Sciences, Tehran, Iran; 3 Department of Radiology, Advanced Diagnostic and Interventional Radiology Research Center, Imam Khomeini Hospital, Tehran University of Medical Sciences, Tehran, Iran; 4 Department of Radiology, Arash Women’s Hospital, Tehran University of Medical Sciences, Tehran, Iran; 5 Department of Surgery, Arash Women’s Hospital, Tehran University of Medical Sciences, Tehran, Iran

**Keywords:** Aspirin, Metformin, Mammographic Breast density, Risk factor

## Abstract

**Background:**

Our purpose was to investigate the association between Mammographic breast density (MBD), a known strong marker for breast cancer and metformin and aspirin use and duration of use alone or simultaneously, in a sample of Iranian women considering other confounding factors.

**Methods:**

In a cross-sectional study, 712 individuals were selected out of women referred to two university hospitals for screening mammography. Participants’ information was collected with a questionnaire. Four-category density scale (a = almost entirely fatty, b = scattered fibroglandular densities, c= heterogeneously dense, and d = extremely dense) was categorized as low (a&b) and high (c&d) density.

**Results:**

The mean age of the participants was 49.80 ± 7.26 years. Sixty-five percent of women belonged to the high and 35% to the low MBD category. Both aspirin and metformin had a significantly negative association with MBD, however, when confounding factors were entered into the models, only aspirin after adjustment for age and BMI had an inverse association with MBD (OR = 0.53, 95% CI: 0.35-0.94). Simultaneous use of metformin and aspirin (OR = 0.44, 95 %CI: 0.17-1.12) was associated with lower MBD. Furthermore, in women who used metformin (OR = 0.23, 95% CI: 0.09-0.62) and aspirin (OR= 0.35, 95% CI: 0.17-0.72) for 2 to 5 years, MBD was significantly lower. However, after the adjustment of confounding factors, these associations were not statistically significant.

**Conclusion:**

It seems metformin and aspirin intakes are associated with MBD. However, further studies with more sample size are needed.

Breast cancer is the most common cancer in women and the incidence is increasing globally. In some of the eastern Mediterranean countries such as Iran, women develop breast cancer at a younger age compared with western countries (1). Preventive therapy for breast cancer with different agents such as selective estrogen receptor modulators and aromatase inhibitors has been shown to reduce the risk of breast cancer by 49% and 65%, respectively (2, 3).

 However, the most important barriers to the wide acceptance and use of these chemoprophylactic agents, especially in healthy women, are their side effects and toxicity (4). 

Therefore, the development of a preventive therapy modality with minimal side effects by the use of more commonly used medicines has been considered. Aspirin and metformin as well-acknowledged, safe profile, low-cost, and multi-action drugs are two likely candidates for the prevention of breast cancer (4). 

Mammographic breast density (MBD) is strongly associated with breast cancer, and a 2-6 fold increased risk of breast cancer is estimated in women with dense breasts compared with those with low MBD (5). However, this association varied between studies and it is not clear whether it is modified by other risk factors. Several studies have examined the effects of metformin and aspirin on breast density with limited confounding factors and have yielded inconsistent findings (6-13). Hence, the present study was designed to further investigate the association between MBD and aspirin and metformin use alone or simultaneously, as well as their duration of regular use, in a sample of Iranian women while considering confounding factors.

## Methods

In this cross-sectional study, individuals were selected from those referred to two university hospitals for screening mammography. Data were collected as part of one study that examined the relationship between environmental factors and breast density (14). That study was approved by the Ethics Committee of Tehran University of Medical Sciences (IR.TUMS.VCR.REC.1398.897) and all participants signed informed consent. Briefly, women who were residents in the capital city of Iran, Tehran, and had the ability to fill out questionnaires, were entered into the study. 

Women who had a prior history of hormone replacement therapy (HRT), breast cancer, or suspicion of malignancy in the current mammography were excluded from this study. Eligible women who were referred to the radiologic center and filled out the questionnaire during a screening mammography appointment were recruited for this study in 2019-2021. 

Furthermore, we gathered information about current aspirin and metformin use and the duration of regular use in each woman. Duration of regular use was categorized as less than 2 years, 2-5 years, and more than 5 years. The MBD in each mammogram was evaluated by two expert radiologists. To evaluate the agreement between the radiologists' reports, the third independent radiologist rated the mammographic breast density of the same cases. MBD was reported according to the American College of Radiology (ACR) Breast Imaging-Reporting and Data System (BI-RADS) four-category density scale (a=almost entirely fatty, b=scattered fibroglandular densities, c=heterogeneously dense, and d=extremely dense) (15). MBD was further categorized into two main classes, low density (a&b) and high density (c&d). 

Cohen's kappa (κ) was run to determine if there was an agreement between two radiologists on breast density in the reports of the same case. A series of multivariable logistic regression models were used to examine the association between aspirin and metformin use and MBD (high breast density versus low density). In each analysis, we fit two models of adjustment: one with all possible effective variables [basis of the association with MBD in univariate analysis (P-value < 0.05)] and one with adjustment of age and BMI. In all models reference group was women who never used aspirin and metformin. The statistical analyses were performed using SPSS Version 26. All tests were two-sided and a p-value less than 0.05 was considered significant. 

## Results

Seven-hundred twelve out of 800 women responded to our questionnaire (89%). The total characteristics of participants were shown in table 1. With almost perfect agreement between the radiologists' report (κ=0.98; p<0.001), 65% (n=463) of women in this study belong to high MBD and 35% (n=249) were in the low MBD category. 

The rate of mammographic low breast density in both drugs users alone or simultaneous is higher than those who did not use it (data are not shown). In analysis with categorized duration use of drugs, breast density was significantly lower in women who used metformin (OR=0.23, 95%CI: 0.09-0.62) and aspirin (OR=0.35, 95%CI: 0.17-0.72) for 2 to 5 years compared with nonusers. Table 2 shows both aspirin and metformin have a significantly reverse association with MBD in univariate analysis, however, only aspirin after adjustment for age and BMI has an inverse association with MBD (OR=0.57, P=0.03).

 Furthermore, we observed a marginally significant lower breast density with simultaneous use of metformin and aspirin (OR=0.44, P=0.08) compared with nonusers. Although in all analyses, after adjustment of covariates, these associations strengthened, were not statistically significant.

**Table 1:** Total characteristics of 712 participants of this study

	**All women** **(n=712)**
**Age (yrs)**	49.80±7.26
**BMI (kg/m** ^2^ **)**	28.16±4.84
**Age at Menarche (yrs)**	13.58±1.53
**Parity (n)**	2.20±1.44
**Breastfeeding (m)**	33.72±29.23
**Menopause**	326(45.8)
**Exposure to smoke**	79(11.1)
**History of OCP use**	262(36.8)
**Calcium**	289(40.6)
**Vitamin D**	352(49.4)
**BC/OC in relatives**	203(28.5)
**MBD** 1 2 3 4	91(12.8) 158(22.2) 427(60) 36(5)
**Metformin users**	75(10.5)
**Aspirin users**	93(13.1)
**Metformin & Aspirin users**	23(3.2)

Data are presented as mean ± standard deviation or number (%). BMI=Body Mass Index; OCP=Oral Contraceptive Pills; BC=Breast Cancer; OC=Ovarian Cancer; MBD=Mammographic Breast Density

**Table 2:** Logistic regression results for models with aspirin, metformin, two agents use simultaneously considering confounding factors

	**OR**	**95% CI**	**P-value**
**Aspirin use (Yes/No)**			
Univariate analysis	0.41	0.26-0.63	**<0.001**
*Model 1*	0.63	0.38-1.04	0.07
*Model 2*	0.57	0.35-0.94	**0.03**
**Metformin use (Yes/No)**			
Univariate analysis	0.54	0.34-0.88	**0.01**
*Model 1*	0.97	0.56-1.70	0.93
*Model 2*	0.90	0.52-1.54	0.70
**Aspirin and metformin use simultaneously (Yes/non-users)**			
Univariate analysis	0.44	0.17-1.12	0.08
*Model 1*	0.91	0.28-2.95	0.87
*Model 2*	0.68	0.24-1.97	0.48

OR=Odds ratio; CI=Confidence interval. Model 1 adjusted for age (as a continuous variable), body mass index (BMI, as a continuous variable), parity (as a continuous variable), oral contraceptive pills usage (OCP, Yes/No), menopausal status (postmenopausal/premenopausal), exposure to smoke (Yes/No), and vitamin D (current continuous user/ non-user). Model 2 adjusted for age and BMI.

## Discussion

The results of our study showed that women taking aspirin or metformin had lower mammographic breast densities. Furthermore, the use of both medicines at the same time was associated with lower density. However, after adjustment for confounding factors, the relationships were not statistically significant. Duration of regular use and daily intake dose of aspirin or metformin may have an impact on breast density and should be considered. Interestingly, in our sample population, intermediate (2 to 5 years) use of aspirin or metformin was associated with lower MBD. Although after adjustment of confounding factors, this association was not statistically significant. 

Similar to our study, two other studies confirmed that short time (less than 2 years) consumption of aspirin and other NSAIDs had not any impact on breast density reduction (6, 7). Another study did not find any association between neither dense areas nor percent dense area with aspirin use at any time after adjustment of variables (8). Other studies had different results regarding the dose or duration of aspirin intake (7, 10, 13).

Epidemiological studies confirmed that the use of metformin in diabetic women was associated with lower MBD, (11, 12) whereas based on Buschard’s study insulin use was associated with an increase in MBD (11). Since several factors such as BMI, reproductive factors, and menopause are associated with breast tissue growth and differentiation, studying these associations by menopausal status could provide clues to the relevance of timing of exposures to changes in breast tissue. The use of vitamin D has evaluated in several studies with controversial results (16, 17) and the association of this supplement and MBD remains poorly understood. Due to the low exposure to sunlight and nutritional and cultural factors, vitamin D level is very low in the Iranian women's community (18) and as our results show, more than 40% of Iranian women take vitamin D supplement regularly (table 1). In the present study, vitamin D intake was associated with increased breast density (OR=1.38, 95%CI: 1.02-1.89). Therefore, in the final analysis of the effect of aspirin and metformin on breast density, we also considered it as confounding factors. 

The present study, for the first time in Iran, evaluated the effect of the current use of two agents (aspirin and metformin) on women's MBD by considering reproductive factors and supplement intake. Our study had some limitations. First, we did not consider the intake dose of aspirin and metformin and could not assess the dose-response in our study. Furthermore, since women consume different brands of drugs; it was not possible to accurately check the effect of any brand in this study. The second limitation was about the sample size of the present study which made us unable to do a sub-analysis regarding some factors such as menopausal status. Finally, our evaluation of breast density was objectively performed by radiologists and automated objective density analysis software was not available. 

It seems metformin and aspirin intakes are associated with decreasing MBD, however, these associations are diminished after considering other influential variables. Of course, simultaneous use of medicines by the individual should also be evaluated when making decisions about preventive medical therapy for breast cancer, due to possible synergistic or antagonistic interaction between them. Considering all hormonal, familial, and environmental issues factors in a context, although necessary, is not possible and each study can evaluate some but not all the variables; this is an essential limitation of these studies. More research is needed to confirm the mechanism of action of these medicines on breast density.
